# Supplementary material for: Localisation-to-delocalisation transition of moiré excitons in WSe2/MoSe2 heterostructures
Source: Nat Commun. 2024 Feb 5;15:1057. doi: 10.1038/s41467-024-44739-9 (PMC10844653; doi:10.1038/s41467-024-44739-9)
Supplement: Supplementary file 1 — Supplementary Information [file 41467_2024_44739_MOESM1_ESM.pdf]

SUPPLEMENTARY INFORMATION for

Localisation-to-delocalisation transition of moiré excitons in $WSe_2/MoSe_2$ heterostructures

Elena Blundo,[1,*] Federico Tuzi,[1] Salvatore Cianci,[1] Marzia Cuccu,[1] Katarzyna Olkowska-Pucko,[2] Łucja Kipczak,[2] Giorgio Contestabile,[1] Antonio Miriametro,[1] Marco Felici,[1] Giorgio Pettinari,[3] Takashi Taniguchi,[4] Kenji Watanabe,[5] Adam Babiński,[2] Maciej R. Molas,[2] and Antonio Polimeni[1,*]

[1] *Physics Department, Sapienza University of Rome, 00185, Roma, Italy.*
[2] *Institute of Experimental Physics, Faculty of Physics, University of Warsaw, Pasteura 5, 02-093 Warsaw, Poland*
[3] *Institute for Photonics and Nanotechnologies (CNR-IFN), National Research Council, 00133, Rome, Italy*
[4] *International Center for Materials Nanoarchitectonics, National Institute for Materials Science, 1-1 Namiki, Tsukuba 305-0044, Japan.*
[5] *Research Center for Functional Materials, National Institute for Materials Science, 1-1 Namiki, Tsukuba 305-0044, Japan*

* Corresponding authors: elena.blundo@uniroma1.it, antonio.polimeni@uniroma1.it

Contents

Supplementary Note 1. Micro-photoluminescence spectra of HS1 and HS2 before and after hBN-capping 1

Supplementary Note 2. Moiré period and stacking angle 4

Supplementary Note 3. Time-resolved micro-photoluminescence data 7

Supplementary Note 4. Estimation of photogenerated carrier density 8

Supplementary Note 5. Time-resolved micro-photoluminescence *vs* power at low temperature 11

Supplementary Note 6. Integrated photoluminescence intensity *vs* power density for different temperatures 12

Supplementary Note 7. Integrated photoluminescence intensity of HS2 *vs* power density for different temperatures 14

Supplementary Note 8. Temperature-dependent micro-photoluminescence 17

Supplementary Note 9. g-factor of moiré and free interlayer excitons in HS2 18

Supplementary Note 10. g-factor of the moiré energy levels 20

Supplementary Note 11. Temperature-dependent g-factor of the MX/IX band 22

Supplementary Note 12. Power studies of the MX/IX band at 16 T and low T 24

References 25

Supplementary Note 1. Micro-photoluminescence spectra of HS1 and HS2 before and after hBN-capping

Fig. 1.1 shows a comparison between the μ-PL spectra of both HS1 and HS2 acquired at 6 K and with the same power both before and after capping with h-BN. Indeed, a shift of the MX band can be noticed for HS1 after capping, while no significant difference can be noticed for HS2. It should also be noticed that the MX band is very close in energy for HS1 (after capping) and HS2.

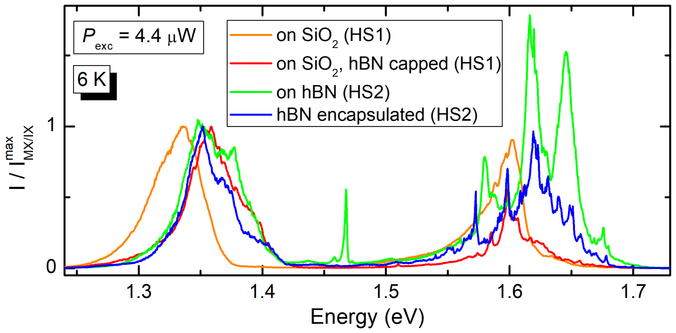

Figure 1.1: μ-PL spectra acquired at 6 K and with analogous power on HS1 (deposited on a SiO_2 substrate) and on HS2 (deposited on a hBN flake) before and after hBN-capping.

The energy of our MX band (E_{PL}) agrees with those reported in the literature. Figure 1.2(a) shows the PL peak energies of $WSe_2/MoSe_2$ HSs with the corresponding reference. All data refer to low temperature (T<20 K) PL measurements and all HSs were encapsulated by h-BN (but papers indicated as g, k and q in the figure legend). We grouped the data into two sets depending

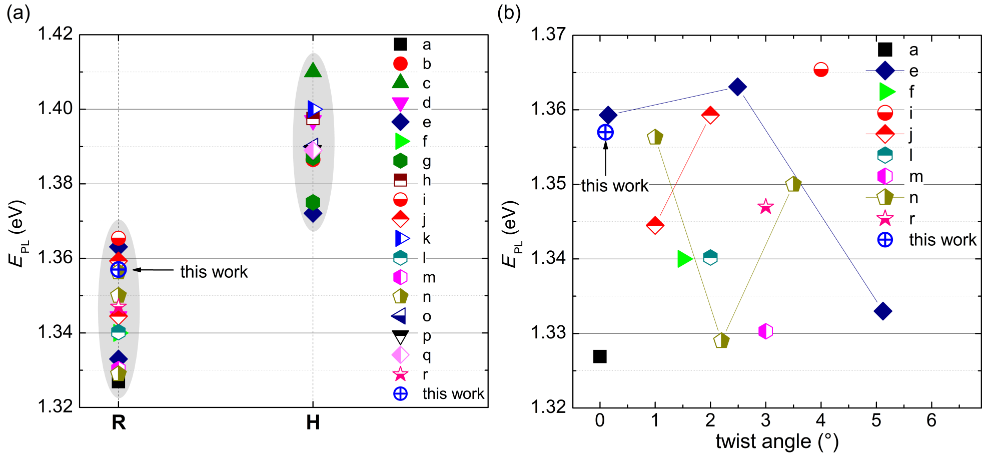

Figure 1.2: (a) Survey of the photoluminescence (PL) peak energies, E_{PL}, of $WSe_2/MoSe_2$ HSs as reported in the literature. R and H indicate HSs with twist angle $\theta\approx$ 0° and $\theta\approx$ 60°, respectively. (b) Same data as in (a) for R-type HSs as a function of θ. The plot shows only data for which the twist angle is provided by the authors. References. a:[1]; b:[2]; c:[3]; d:[4]; e:[5]; f:[6]; g:[7]; h:[8]; i:[9]; j:[10]; k:[11]; l:[12]; m:[13]; n:[14]; o:[15]; p:[16]; q:[17]; r:[18].

on the relative alignment angle: *i*) R-type HSs corresponding to a twist angle, θ, around 0° and *ii*) H-type HSs corresponding to θ around 60°. We note that the E_{PL} values relative to each type of HS remain rather distinct, although a large spread of values is observed within each type of HS. Fig. 1.2(b) shows the dependence on θ of E_{PL} for R-type HSs (namely, the type investigated

in our work). The plot includes E_{PL} values for those works, where θ was given. Clearly, no well-defined trend can be overall observed. This is likely due to the large uncertainties in the determination of θ, local deviations from a regular structure caused by imperfections and local strains accompanied by important atomic level reconstruction that result in a non-ideal/regular moiré potential [19]. The R-type HSs considered in our work feature E_{PL} values, which are higher than the average reported values, yet well within the literature distribution of E_{PL} for R-type HSs (see Fig. 1.2).

As for our HSs, we tested their homogeneity by scanning the laser across their surface.

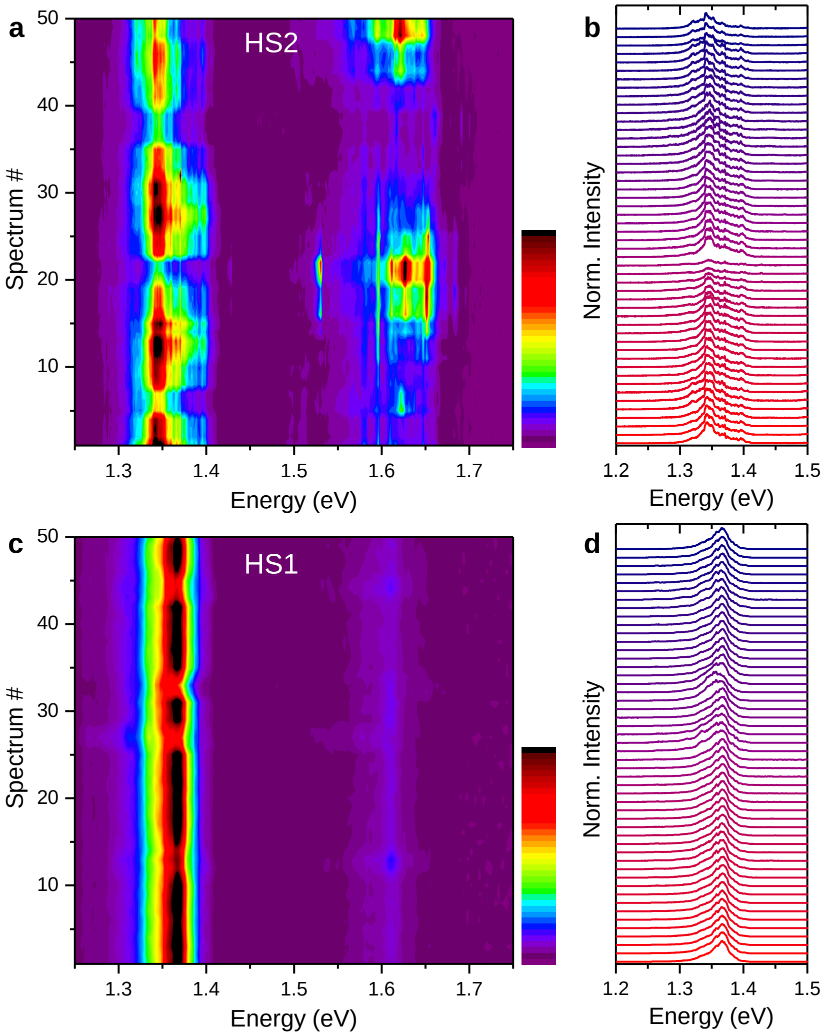

Figure 1.3: a-b Colour plot (**a**) and normalised stacked spectra (**b**) of the μ-PL measurements performed while scanning the laser ($P_{\mathrm{exc}} = 0.22\ \mu$W) over HS2 with an S-like scan. The scanning step was of about 0.3 μm. **c-d** Same for HS1. The drops in the intensity of the MX (and corresponding enhancement of the intralayer excitons signal), which can be noticed especially for HS2, are due to the fact that the HS edge was reached. Apart from such drops, the intensity and lineshape of the MX is pretty uniform.

Fig. 1.3 shows such a study performed on both HS2 and HS1. The measurements were taken by performing an S-scan on the central part of each HS, with steps of about 0.3 μm. The intensity drops revealed by the colour plots are due to the fact that the scan reached the edge of the HS. Overall, both HSs show a very good degree of homogeneity as far as the resonance energy distances and relative spectral weight are concerned. A bit larger degree of fluctuation is instead observed regarding the emission intensity.

Overall, the central part of the HSs is pretty uniform, and some variations can be noticed only towards the edges. Our study reveals that the sample homogeneity is indeed such that the results discussed in our work hold a general validity and are not related to some specific spots in the sample.

Supplementary Note 2. Moiré period and stacking angle

The Hamiltonian for excitons confined in a moiré potential can be described as [20, 10]:

$$H = \hbar\Omega_0 + \frac{\hbar^2 k^2}{2M} + \Delta(\mathbf{r}), \tag{2.1}$$

where the first term is an energy constant, the second term is the center of mass kinetic energy, M is the exciton mass and $\Delta(\mathbf{r})$ is the moiré potential energy. For a $MoSe_2/WSe_2$ HS, $M \approx 0.84 m_e$, where m_e is the electron bare mass [10]. Near its minima, the moiré potential $\Delta(\mathbf{r})$ can be approximated as parabolic: $\Delta(\mathbf{r}) = \beta(\mathbf{r}/a_M)^2/2$, where a_M is the moiré potential period and β is a constant independent of a_M. Excitons confined in this parabolic potential have quantised energy levels:

$$E_m = \sqrt{\frac{\beta\hbar^2}{Ma_M^2}} \cdot (n_x + n_y + 1), \tag{2.2}$$

where $n_{x,y}$ are non-negative integers [20]. The spacing between subsequent levels is thus:

$$S = \sqrt{\frac{\beta\hbar^2}{Ma_M^2}}. \tag{2.3}$$

Following ref. [10], $\beta = 2.84$ eV.

The moiré period a_M can thus be estimated by measuring the spacing, as:

$$a_M = \frac{1}{S} \cdot \sqrt{\frac{\beta\hbar^2}{M}}. \tag{2.4}$$

For our HSs, we thus acquired low power (5 nW) spectra at 6 K with higher spectral resolution (we used a monochromator with a focal length of 75 cm), as shown in Fig. 2.1, and fitted the spectra with 5 gaussians. The number of gaussians was established empirically, in order to reproduce the spectra reliably but to avoid exceeding with their number (and thus with the fitting parameters). We performed the fitting without imposing strict conditions on the spacing, and determined the average spacing for both HS1 and HS2 as: $S_{\mathrm{HS1}} = (12.8 \pm 1.3)$ meV and $S_{\mathrm{HS2}} = (20.3 \pm 3.4)$ meV. Through Eq. 2.4, we estimate: $a_{M,\mathrm{HS1}} = (39.8 \pm 3.9)$ nm and $a_{M,\mathrm{HS2}} = (25.1 \pm 4.2)$ nm. According to the calculations by Liu *et al.* [2], such periods correspond to stacking angles of $\theta_{\mathrm{HS1}} = (0.46^{+0.05}_{-0.04})^\circ$ and $\theta_{\mathrm{HS2}} = (0.74^{+0.16}_{-0.11})^\circ$.

Indeed, these angles are both very close to zero. For HS2, the fabrication process was made based on the crystal orientation derived by second harmonic generation (SHG) measurements. The HS was fabricated with a virtually null twist angle, but the uncertainty in the SHG data and those in the fabrication process itself entail an uncertainty of about a couple degrees in the fabrication process. The estimated angle $\theta_{\mathrm{HS2}} \approx 0.74^\circ$ is thus in good agreement with the expected null angle. For HS1, instead, the fabrication process was not based on SHG measurements. We thus performed SHG *a posteriori* to verify the crystal orientation. The results are displayed in Fig. 2.2.

The SHG measurements were performed while keeping the polarisation of the detected signal and that of the excitation laser fixed and parallel. The sample was then rotated by an angle θ with respect to an arbitrary set of laboratory coordinates (X,Y). Fig. 2.2 displays the SHG measurements taken on pieces of the WSe_2 and $MoSe_2$ MLs that stick out of the HS. The two sets of data were then fitted by the equation:

$$I_{\mathrm{SHG}} = \left[A \cdot \cos(3\theta')\right]^2, \quad \theta' = \theta - \theta_0 \tag{2.5}$$

where θ' is the angle between the excitation laser polarisation and the armchair direction, and θ_0 defines the direction of the armchair lattice direction with respect to the X axis of the laboratory

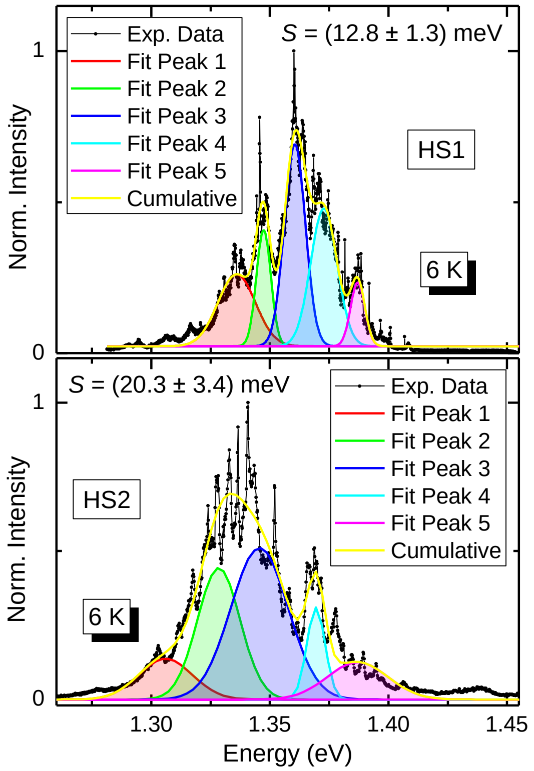

Figure 2.1: μ-PL spectra of the MX band acquired with very low laser power excitation (5 nW). The spectrum can be reproduced by five Gaussian functions that are spaced on average by (12.8±1.3) meV (the individual spacings are 11.4, 13.2, 12.2, 14.3 meV) for HS1, and by (20.3 ± 3.4) meV (the individual spacings are 22.5, 17.1, 23.8, 17.6 meV) for HS2. The very narrow lines that make up the broader Gaussian peaks correspond to single MXs recombining in moiré minima.

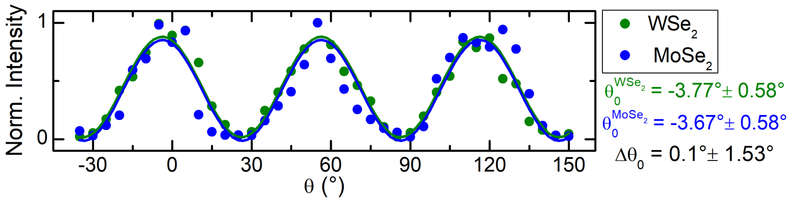

Figure 2.2: SHG normalised intensity measured in the WSe_2 and $MoSe_2$ MLs that constitute HS1 as a function of the rotation angle of the sample.

system. Indeed, the θ_0 angles found for WSe_2 and $MoSe_2$ are very close to each other, with a relative twist $\Delta\theta_0 = 0.1° \pm 1.53°$. This confirms that our HS is characterised by a very small twist angle close to zero, in agreement with the results obtained by the analysis of the spacing between the enregy levels of the moiré band.

Finally, we also performed a comparative study between the decay times of the PL signal of the two HSs. Indeed, Choi et al. [14] showed how the decay time of MX/IX is sensitive to the twist angle, and in particular increases dramatically while going from a twist of 1° to 3°, a feature related to the larger k-space mismatch for larger twist angles. Indeed, as shown in Fig.2.3 , HS2 exhibits exciton PL decay times longer than those observed in HS1 as expected based on the slightly larger twist angle of HS2 with respect to HS1.

Our results agree very well with those reported in Ref. [14] for a $MoSe_2/WSe_2$ HS with a 1° twist angle, for which a 21 meV spacing was found between the energy levels (thus very close to what we found for HS2). In that work, the time-resolved measurements concerning the HS with

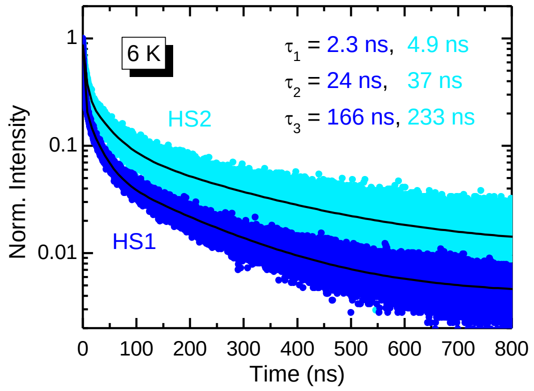

Figure 2.3: Time-evolution of the μ-PL signal of HS2 recorded in the Δt=0-800 ns interval from the laser pulse and comparison with the analogous data acquired for HS1. The excitation power was 1 μW. The data were fitted by Eq. 1 of the main text, and the estimated decay times are displayed, showing how larger decay times are found for HS2 compared to HS1.

a 1° twist angle were collected only in the (0-25) ns range from the laser pulse and were fitted by a double exponential function providing decay times of 1-2 ns and 6-10 ns. If we restrict our data to the same time range of (0-25) ns and fit them with a double exponential, we get decay times of about 2 and 11 ns for HS2, which indeed agree well with those by Choi et al. [14]. This discussion highlights however the importance of acquiring and analysing the time-resolved data on an appropriate range, since when restricting the data to a relatively short range the result is possibly much more affected by the presence of non-radiative decay channels and the slow dynamics that characterises MXs cannot be fully appreciated.

Supplementary Note 3. Time-resolved micro-photoluminescence data

Fig. 3.1 shows the time resolved μ-PL data of Fig. 2 of the main text, displayed on a suitable time scale in order to compare them with the laser pulse data. Indeed, the data concerning the

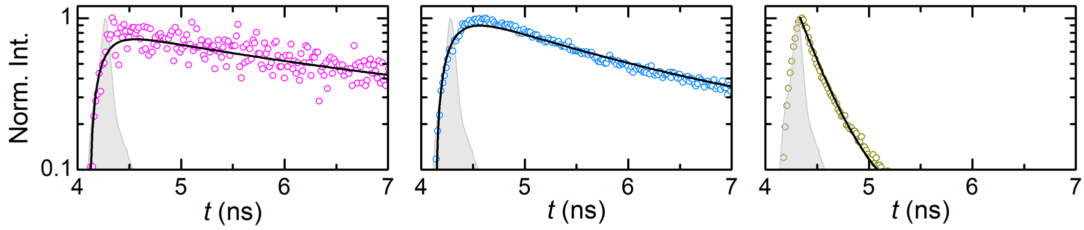

Figure 3.1: Time-evolution of the μ-PL signal and laser pulse (light grey) on the three spectral regions highlighted in Fig. 2**a** of the main text. The data are the same of Figs. 2**b-c** of the main text, but plotted in the first few ns of acquisition in order to enable a comparison with the laser curve.

low (pink) and middle (cyan) energy ranges, show a much slower rise and decay dynamics if compared to the laser pulse. For the high energy range, instead, the decay part gets faster (but still longer than the resolution limit), while the rise part of the data is clearly resolution limited (in fact, as discussed in the main text, the rise time cannot be derived).

By fitting the decay part of the data in Fig. 2**b** of the main text, we obtained the fitting weights and decay times displayed in the following table.

Table 3.1: Fitting parameter values obtained by fitting the data in Fig. 2**b** by Eq. (1) of the main text. $\tau_{\mathrm{d,n}}$ is the decay time relative to the n-th component, whose weight is given by $w_{\mathrm{d,n}}$.

Energy Range	$\tau_{\mathrm{d,1}}$ (ns)	$w_{\mathrm{d,1}}$ (%)	$\tau_{\mathrm{d,2}}$ (ns)	$w_{\mathrm{d,2}}$ (%)	$\tau_{\mathrm{d,3}}$ (ns)	$w_{\mathrm{d,3}}$ (%)
Low (pink)	2.27 ± 0.09	60.9 ± 1.4	14.0 ± 0.6	34.6 ± 0.8	51 ± 4	4.4 ± 0.7
Medium (cyan)	1.59 ± 0.01	78.1 ± 0.4	15.2 ± 0.2	15.8 ± 0.1	77.0 ± 0.3	6.0 ± 0.1
High (yellow)	< 0.23	86.0 ± 0.4	1.03 ± 0.01	13.0 ± 0.3	13.5 ± 0.1	1.0 ± 0.1

Supplementary Note 4. Estimation of photogenerated carrier density

Figure 3 in the main text shows the cw μ-photoluminescence (PL) spectra at T=6 K varying the laser power $P_{\rm exc}$ between 44 nW and 100 μW. To each $P_{\rm exc}$ value we associate a specific density of electron-hole pairs $n_{\rm e-h}$ photogenerated within the HS. This was done following Ref. [9]. Below we summarise the procedure followed.

Under continuous wave (cw) excitation, the generation rate of photogenerated carriers is given by

$$G(n_{\rm e-h}) = \frac{P_{\rm exc} \cdot \sigma(n_{\rm e-h})}{S \cdot h\nu}, \tag{4.1}$$

where $\sigma(n_{\rm e-h})$ is the dependence of the absorbance of the $MoSe_2/WSe_2$ HS on the photogenerated carrier density, $S = \pi r^2$ is the laser spot area with r=500 nm, and hν=2.33 eV is the exciting photon energy. In addition, in a stationary (*i.e.* continuous wave) regime we have

$$\frac{{\rm d}n_{\rm e-h}}{{\rm d}t} = G(n_{\rm e-h}) - \frac{n_{\rm e-h}}{\tau(n_{\rm e-h})} = 0, \tag{4.2}$$

where $\tau(n_{\rm e-h})$ is the dependence of the exciton decay time of the $MoSe_2/WSe_2$ HS on the photogenerated carrier density. To solve this equation we need to derive $\sigma(n_{\rm e-h})$ and $\tau(n_{\rm e-h})$.

$\sigma(n_{\rm e-h})$ was previously reported in Ref. [9] and it is reproduced in Fig. 4.1.

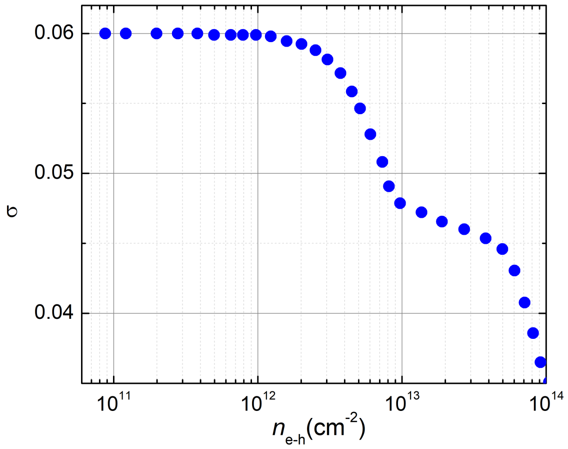

Figure 4.1: Optical absorbance of a $MoSe_2/WSe_2$ HS as a function of the density of photogenerated carriers for photon energy equal to 2.33 eV. The data were taken from Fig. 4 of Ref. [9].

$\tau(n_{\rm e-h})$ was deduced in two steps. We first measured the time decay of the exciton μ-PL signal, whose temporal traces are shown in Fig. 4.2 for different $P_{\rm exc}$s. We then fitted the data using Eq. (1) in the main text with $n = 3$ components characterised by their decay time $\tau_{\rm d,n}$ with relative weight $w_{\rm d,n}$. Table 4.1 reports the $\tau_{\rm d,n}$ and $w_{\rm d,n}$ values for different $P_{\rm exc}$s along with the weighted value of the decay time τ. Finally, we obtained $\tau(n_{\rm e-h})$ using the following relationship under pulsed excitation between the injected carrier density n and $P_{\rm exc}$:

$$n = \frac{P_{\rm exc} \cdot \sigma}{S \cdot f_{\rm rep} \cdot h\nu}, \tag{4.3}$$

where σ=0.08 is the absorbance of the $MoSe_2/WSe_2$ HS evaluated for hν=2.33 eV as reported in Ref. [9] of this Supplementary Material, and $f_{\rm rep}$=1.2 MHz is the pulsed laser repetition rate. Therefore, we can relate n to $P_{\rm exc}$ and hence to τ via Table 4.1. Fig. 4.3 shows the dependence of τ on n.

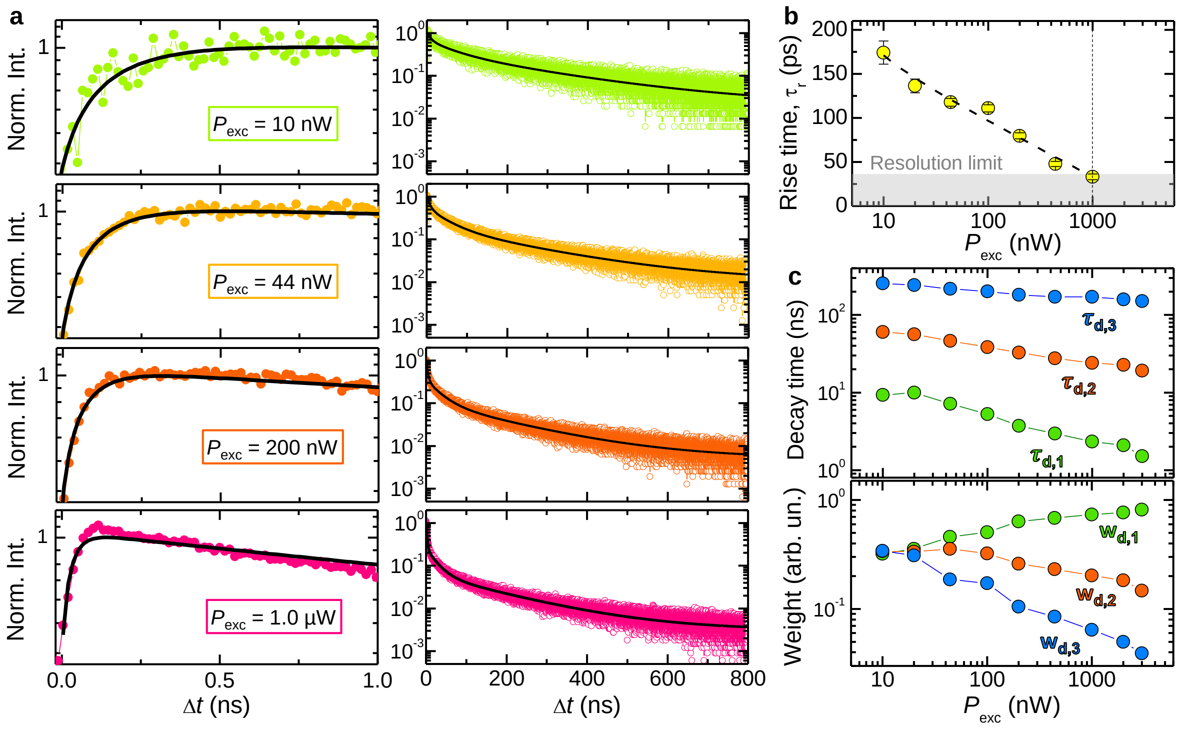

Figure 4.2: **a** Some exemplifying plots of the time-evolution of the μ-PL signal of the investigated $WSe_2/MoSe_2$ HS recorded at 6 K for different excitation powers P_{exc}, in the Δt=0-800 ns interval from the laser pulse. The detection energy was set at the MX-IX band. The solid lines are fit to the data by Eqs. (2) (left column) and (1) (right column) of the main text. **b** Summary of the estimated rise times τ_r as a function of P_{exc}. Above 1 μm (as highlighted by the vertical dashed line) the rise time goes below the resolution limit of our setup. **c** Summary of the estimated decay times $\tau_{d,n}$ as a function of P_{exc} and corresponding weights $w_{d,n}$.

Table 4.1: Fitting parameter values shown in Fig. 4.2**b**. $\tau_{d,n}$ is the decay time relative to the n-th component, whose weight is given by $w_{d,n}$. The last column reports the weighted decay time τ.

P_{exc} (nW)	$\tau_{d,1}$ (ns)	$w_{d,1}$	$\tau_{d,2}$ (ns)	$w_{d,2}$	$\tau_{d,3}$ (ns)	$w_{d,3}$	τ (ns)
10	9.3	0.32	60.2	0.34	254.9	0.34	110.4
20	10	0.36	56.2	0.34	242.0	0.31	97.1
44	7.2	0.46	46.3	0.36	216.4	0.19	60.0
100	5.3	0.50	38.4	0.32	201.2	0.17	49.9
200	3.7	0.64	32.7	0.26	180.9	0.10	29.8
440	3.0	0.68	27.5	0.23	171.4	0.08	22.9
1000	2.3	0.73	24.1	0.20	170.8	0.06	17.6
2000	2.1	0.77	22.6	0.18	159.2	0.05	13.7
3000	1.5	0.81	19.1	0.15	150.7	0.04	9.9

Given the results shown in Figs. 4.1 and 4.3, we can solve Eq. 4.3 –namely n_{e-h}=$G(n_{e-h}) \cdot \tau(n_{e-h})$– numerically as displayed in Fig. 4.4. The solutions are given by the crossing of the first and second member of the above equation plotted on the y and x axis, respectively. That plot finally allows us to give the density of photogenerated electron-hole pairs for a specific excitation power P_{exc}.

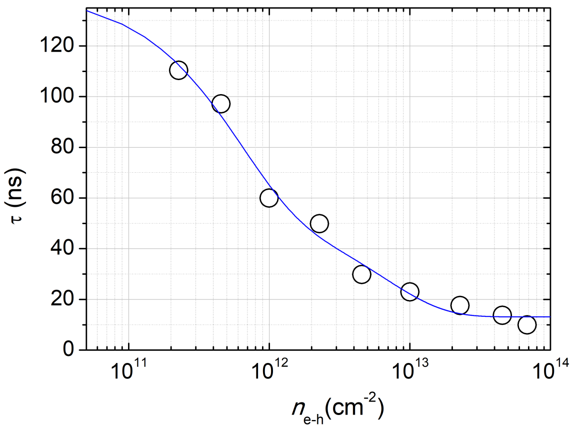

Figure 4.3: Dependence of the weighted decay time τ (symbols; see Table 4.1) on the density of photogenerated carriers n_{e-h}. The solid line is a bi-exponential fit used to interpolate the data.

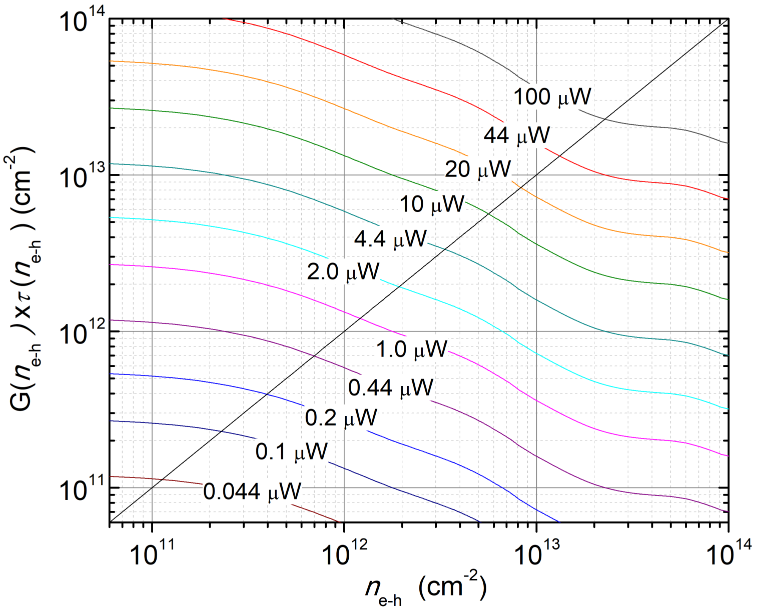

Figure 4.4: Plot of $G(n_{e-h}) \cdot \tau(n_{e-h})$ *vs* n_{e-h}. The intersections between the bisecting black line and the other curves gives the solutions of Eq. 4.2 for each of the cw P_{exc} values considered in Fig. **3a** of the main text.

Supplementary Note 5. Time-resolved micro-photoluminescence *vs* power at low temperature

Analogous measurements to those discussed in Supplementary Note 4 for HS1 were performed also for HS2. In particular, we performed time-resolved μ-PL measurements at 6 K by varying the excitation power over more than two orders of magnitude. The decay times and corresponding weights estimated for HS1 and HS2 and displayed and compared in Fig. 5.1. Indeed, very similar

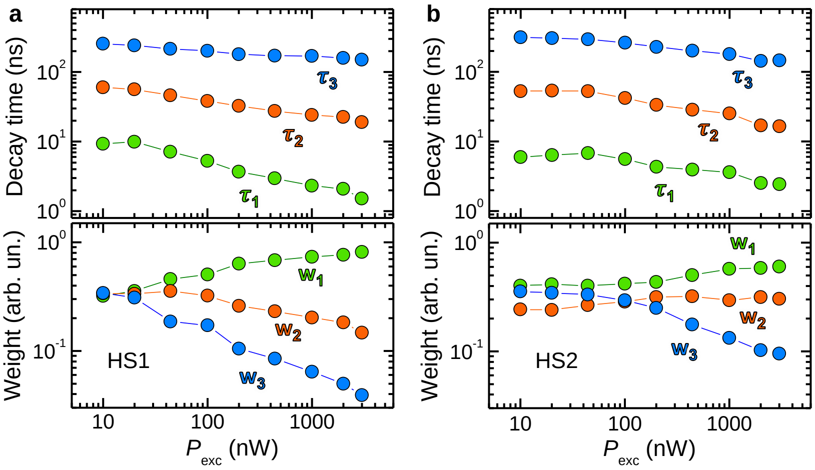

Figure 5.1: Decay times and weights derived from time-resolved μ-PL measurements performed at 6 K for different excitation powers P_{exc}. The data were acquired in the Δt=0-800 ns interval from the laser pulse. The detection energy was set at the MX-IX band. **a** Summary of the estimated decay times $\tau_{d,n}$ as a function of P_{exc} (top) and corresponding weights $w_{d,n}$ (bottom), for HS1. **b** Same for HS2.

trends are observed for both HS1 and HS2, with HS2 generally showing slightly slower decay times due to the slightly larger twist angle (see Supplementary Note 2).

Supplementary Note 6. Integrated photoluminescence intensity of HS1 *vs* power density for different temperatures

The data shown in Fig. 3**c** of the main text were derived by fitting the PL integrated intensity I of HS1 at different temperatures using the following equation

$$I = A \cdot P_{\mathrm{exc}}^{\alpha}, \tag{6.1}$$

where P_{exc} is the laser excitation power, A is a scaling constant and α a coefficient, whose value suggests the type of transition involved (*e.g* α=1 exciton transition, α=2 uncorrelated electron-hole pairs, α<1 finite density two-level system). Figure 6.1 shows the dependence of I on P_{exc} for the two main bands observed MX-IX (interlayer exciton, either moiré, MX, or free, IX) and X (free exciton of the heterostructure constituents); see, *e.g.* Figs 2 (a) and 3 (a) in the main text. The measurements were performed at different temperatures between 6 K and 296 K. The results of the fits are displayed in the different panels of Figure 6.1.

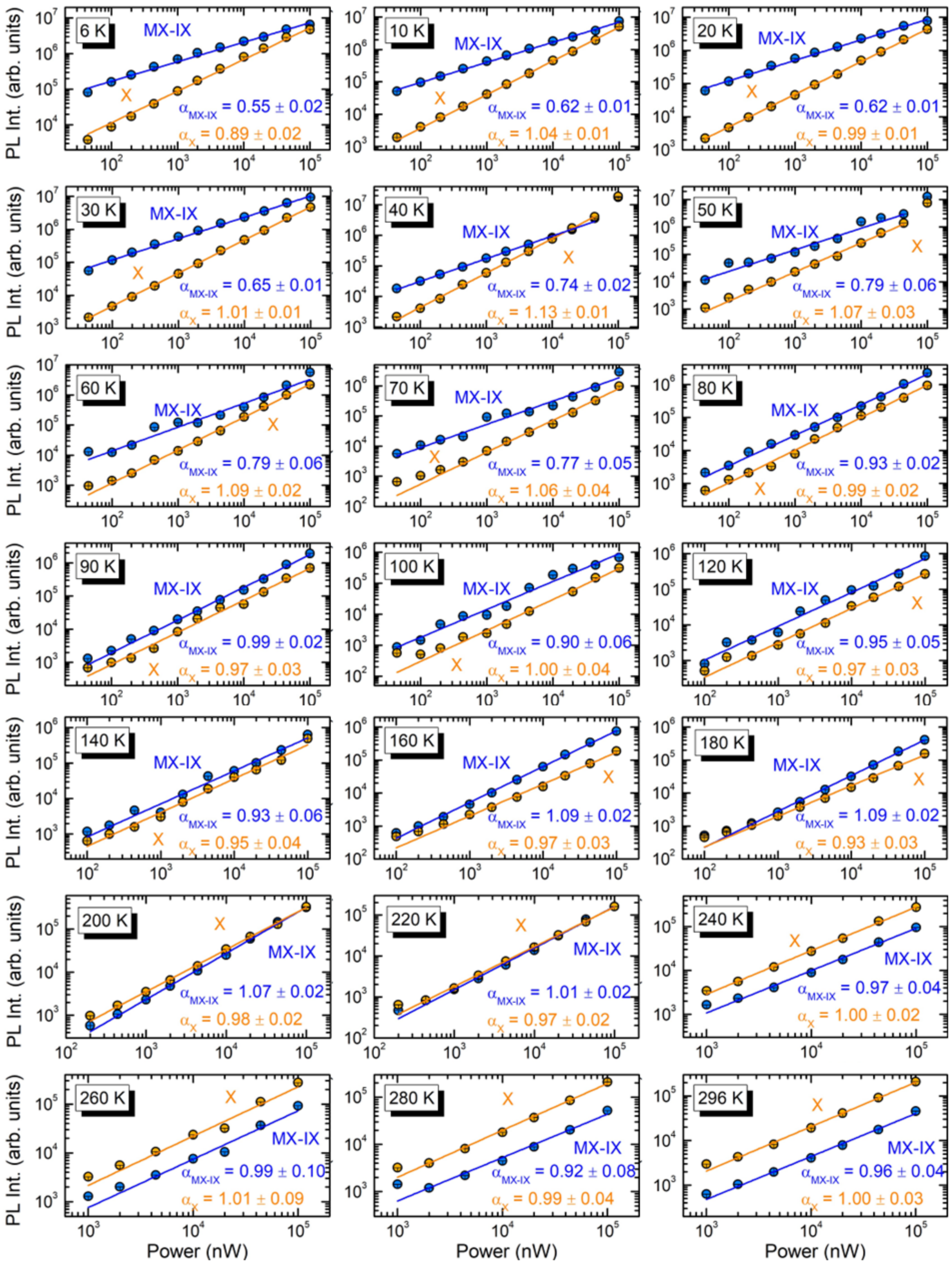

Figure 6.1: PL integrated intensity dependence on the laser power for MX-IX (azure symbols) and X (dark yellow symbols) bands at different temperatures for HS1. Solid lines are fits to the data via Eq. 6.1. The α coefficient values obtained from the fits are displayed within each plot.

Supplementary Note 7. Integrated photoluminescence intensity of HS2 *vs* power density for different temperatures

Fig. 3 of the main text and Supplementary Note 6 clearly show the transition from a localised to a delocalised regime for the interlayer exciton when increasing temperature, which is evidenced by power-dependent μ-PL studies on HS1. To ensure the general validity of our statements, we repeated analogous measurements on HS2. As noted in Fig. 3**d** of the main text, at intermediate temperatures of about 90 K a dramatic lineshape change can be observed when increasing power, showing a clear transition from the MX prevailing at low $P_{\rm exc}$ to the IX prevailing at high $P_{\rm exc}$. The same trend was indeed observed also for HS2, as demonstrated by the power-dependent spectra acquired at 80 K and shown in Fig. 7.1.

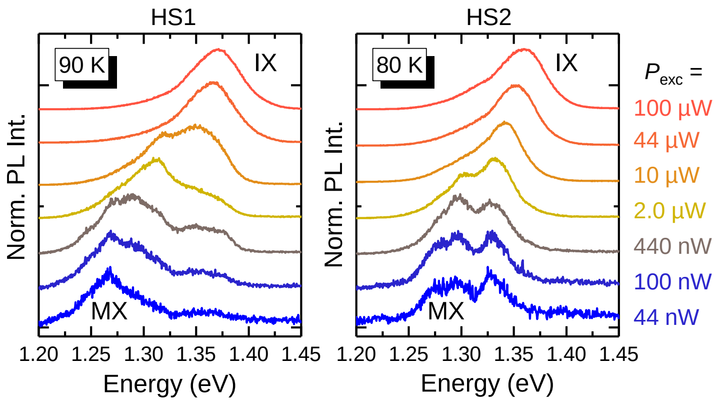

Figure 7.1: Left: T=90 K μ-PL spectra of HS1 for different laser excitation powers in the energy region where the MX and IX recombinations can be simultaneously observed. IX takes over MX upon increase of the photo-generated carrier density. Right: Same at T=80 K for HS2.

Power-dependent measurements were performed at different temperatures between 6 K and 296 K, analogously to HS1. The integrated PL intensity of the MX/IX and of the X band were then analysed and fitted by Eq. 3 of the main text, leading to the α coefficients shown in Fig. 7.2.

Similarly to HS1, also for HS2 one can notice a saturation behaviour (evidenced by the sublinear power trend, *i.e.*, $\alpha < 1$) of the MX/IX band at low temperatures, with the α coefficient that increases with temperature. From about 100 K, the power trend becomes linear ($\alpha = 1$). Interestingly, for HS2 the linear trend is reached at a T value about 20 K lower than for HS1 (as highlighted by the black dashed lines is Fig. 7.2), consistently with the shallower moiré potential expected for larger twist angles.

The whole set of data concerning the dependence of the PL integrated intensity I of HS2 on $P_{\rm exc}$ for the MX-IX and X bands and the corresponding fits, from which the α coefficients were derived, is shown in Fig. 7.3.

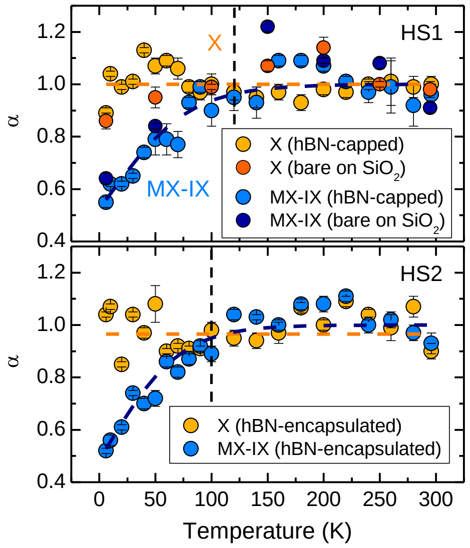

Figure 7.2: Temperature variation of the α coefficient for the MX-IX and X bands, comparison between HS1 and HS2. For HS1, some points measured before hBN-capping were also included in the plot, showing that the data agree well with those acquired after hBN-capping. Both for HS1 and HS2, a clear transition from a sublinear to a linear behaviour is found for the MX-IX band, which is ascribed to the transition from a moiré localisation regime to a free interlayer exciton one. Such a transition occurs at ≈ 120 K for HS1, and for ≈ 100 K for HS2, as highlighted by the black dashed lines.

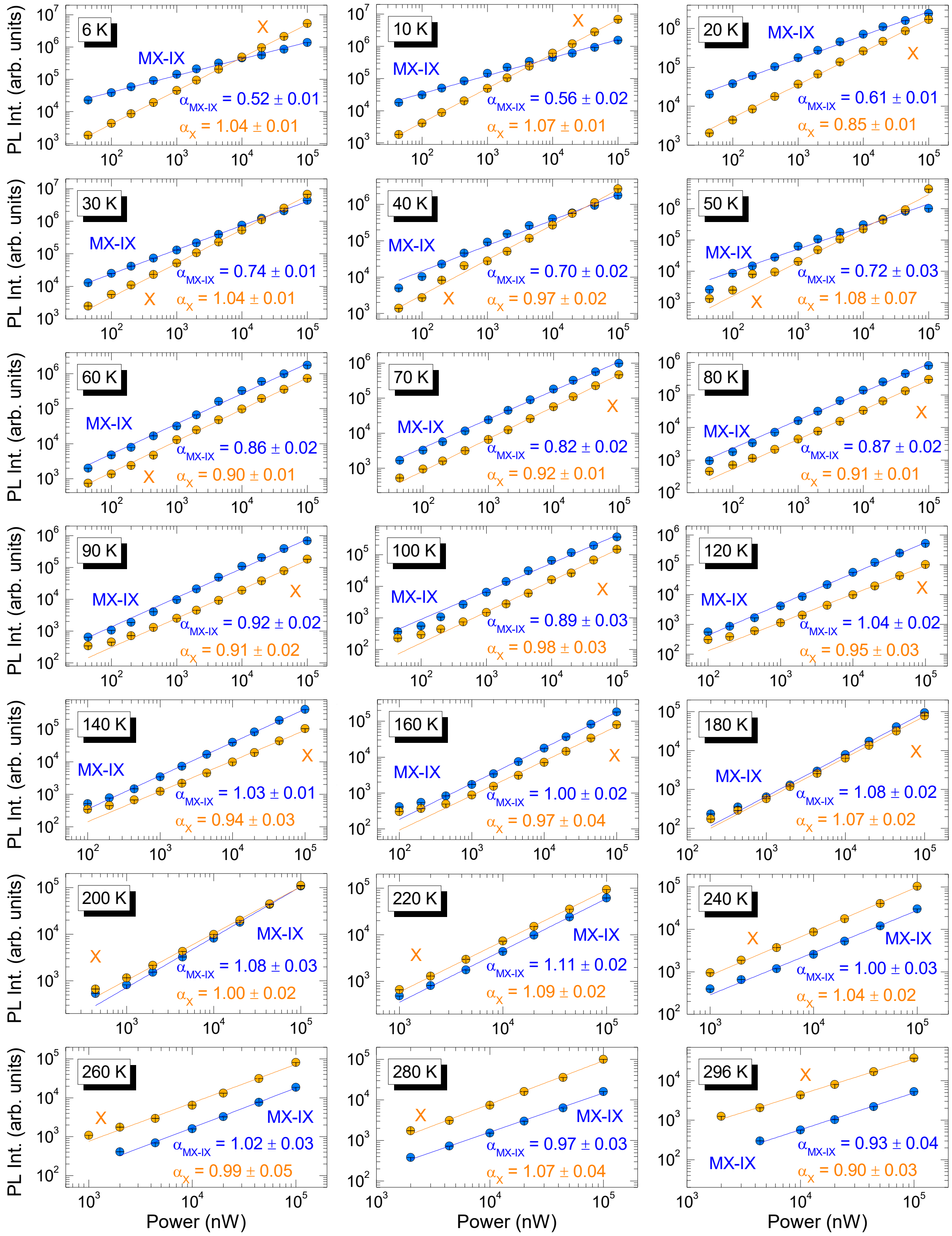

Figure 7.3: PL integrated intensity dependence on the laser power for MX-IX (azure symbols) and X (dark yellow symbols) bands at different temperatures for HS2. Solid lines are fits to the data via Eq. 6.1. The α coefficient values obtained from the fits are displayed within each plot.

Supplementary Note 8. Temperature-dependent micro-photoluminescence

Figure 4**a** in the main text shows the temperature dependence of the μ-photoluminescence (PL) spectra recorded on the investigated WSe_2/$MoSe_2$ heterostructure for a given laser excitation power P_{exc} (=10 μW). The set of data shows a clear T-induced variation in the emission lineshape caused by the de-trapping of moiré-localised excitons (MXs) in favour of free interlayer excitons (IXs). Figure 8.1 shows a similar study performed at a higher (=100 μW) and lower (=1 μW) P_{exc}. Figure 8.1 indicates that, for a given T, the de-trapping process becomes more apparent for a larger density of photogenerated carriers (*i.e.* larger P_{exc}).

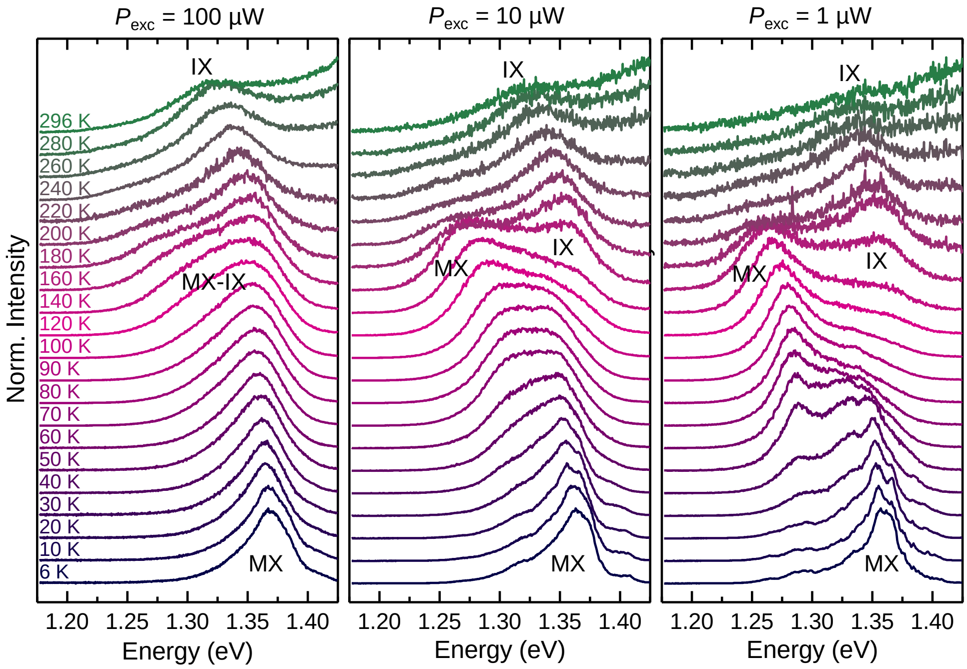

Figure 8.1: μ-PL spectra *vs* T at three different laser excitation powers P_{exc} (focused via a 20× objective with NA = 0.4). MX and IX indicate the recombination band due to moiré-localised excitons (MXs) and free interlayer excitons (IXs), respectively.

This can be better appreciated in Figure 8.2. Indeed, for fixed T=160 K, the relative weight of the IX component increases for increasing number of photogenerated carriers and for the ensuing saturation of the finite moiré potential sites. This saturation takes place more evidently as T increases, which favours moiré de-trapping as shown in the previous figure.

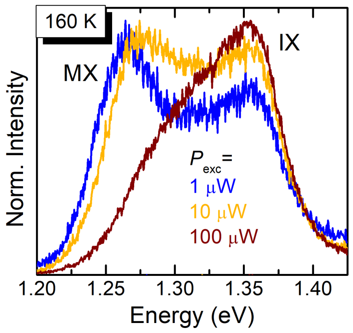

Figure 8.2: μ-PL spectra at 160 K for different laser excitation powers P_{exc} (focused via a 20× objective with NA = 0.4). MX and IX indicate the recombination band due to moiré-localised excitons (MXs) and free interlayer excitons (IXs), respectively.

Supplementary Note 9. g-factor of moiré and free interlayer excitons in HS2

Fig. 4 of the main text shows the result of magneto-μ-PL measurements performed on HS1 at low power and low T —to measure the g-factor of MX lines— and at high power and high T —to measure the g-factor of free interlayer excitons. To ensure the general validity of our results, we performed similar measurements on HS2. Fig. 9.1**a** shows the polarisation-resolved magneto-μ-PL spectra acquired on HS2 at 10 K with $P_{\text{exc}} = 50$ nW, while varying the magnetic field from 0 to 16 T in steps of 0.5 T. While the field increases, the energy of the narrow lines (see, *e.g.*, lines denoted as M1 and M2) is higher for the σ^+ polarisation with respect to the σ^- one, and the g-factor is thus positive. Fig. 9.1**b** shows instead the spectra acquired at 160 K with $P_{\text{exc}} = 100$ μW. The spectra were acquired while increasing the field from 0 to 12 T in steps of 0.5 T (only some spectra are shown in the figure for ease of visualisation). In this case, the IX can be observed, and the σ^- component lies at higher energy than the σ^+ component, indicating a negative g-factor.

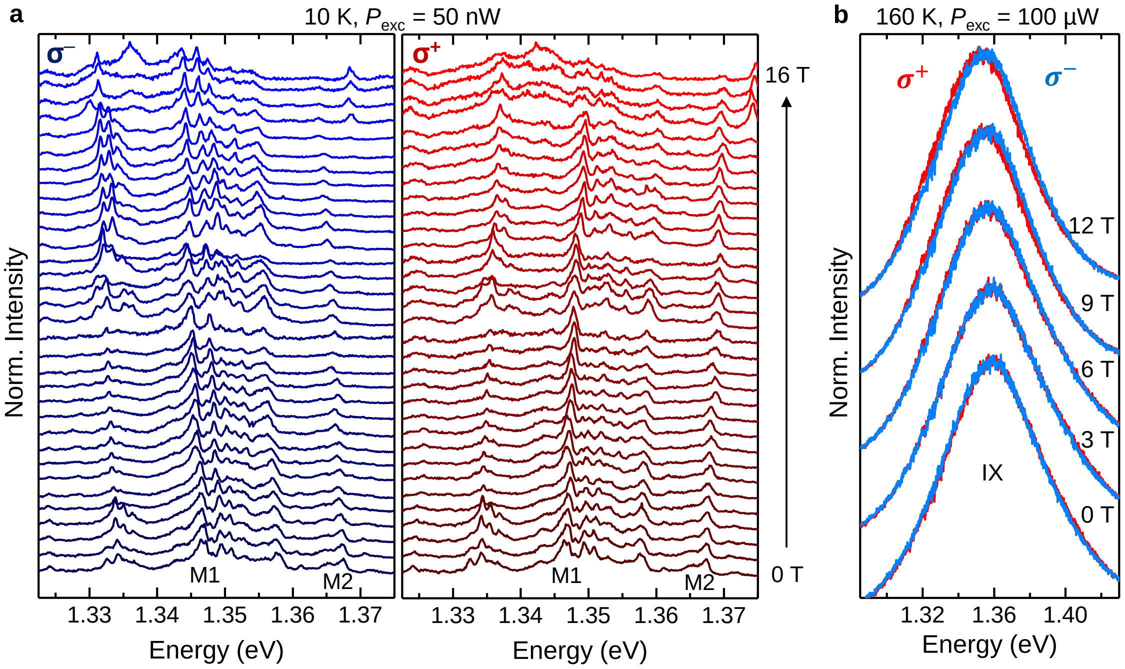

Figure 9.1: Magneto-μ-PL measurements on HS2 at low and high Ts. **a** Polarisation-resolved magneto-μ-PL spectra acquired on HS2 with $P_{\text{exc}} = 50$ nW from 0 to 16 T in steps of 0.5 T. Two moiré narrow lines (Ms) are indicated. **b** Polarisation-resolved magneto-μ-PL spectra at $T = 160$ K and P_{exc}=100 μW of the free IX band for σ^+ and σ^- polarisations. A negative ZS can be observed, with the σ^+ and σ^- spectra being at lower and higher energy, respectively.

A quantitative analysis of the g-factor of the moiré narrow lines M1 and M2 at 10 K and of the IX band at 160 K is shown in Fig. 9.2. The g-factor values estimated for HS2 are indeed very close to those found for HS1 and displayed in Fig. 4**d** of the main text.

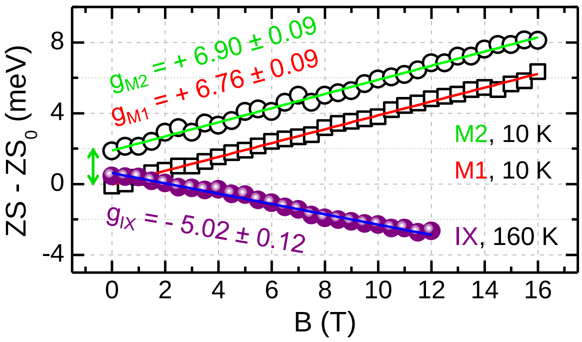

Figure 9.2: g-factor of the MXs and IX of HS2. **d** ZS of the two moiré-localised excitons M1 and M2 highlighted in Fig. 9.1**a** and of the free IX exciton shown in Fig. 9.1**b** *vs* magnetic field, resulting in the g-factors displayed in the figure. The ZS data of the M2 line was shifted by y-offset (by 2 meV) for ease of visualisation.

Supplementary Note 10. g-factor of the moiré energy levels

Fig. 10.1 shows the polarisation-resolved magneto-μ-PL spectra acquired on HS1 as a function of the magnetic field, with excitation powers as low as 10 nW. The data shown here are the complete set of data from which the plots in Fig. 4**b** of the main text were derived.

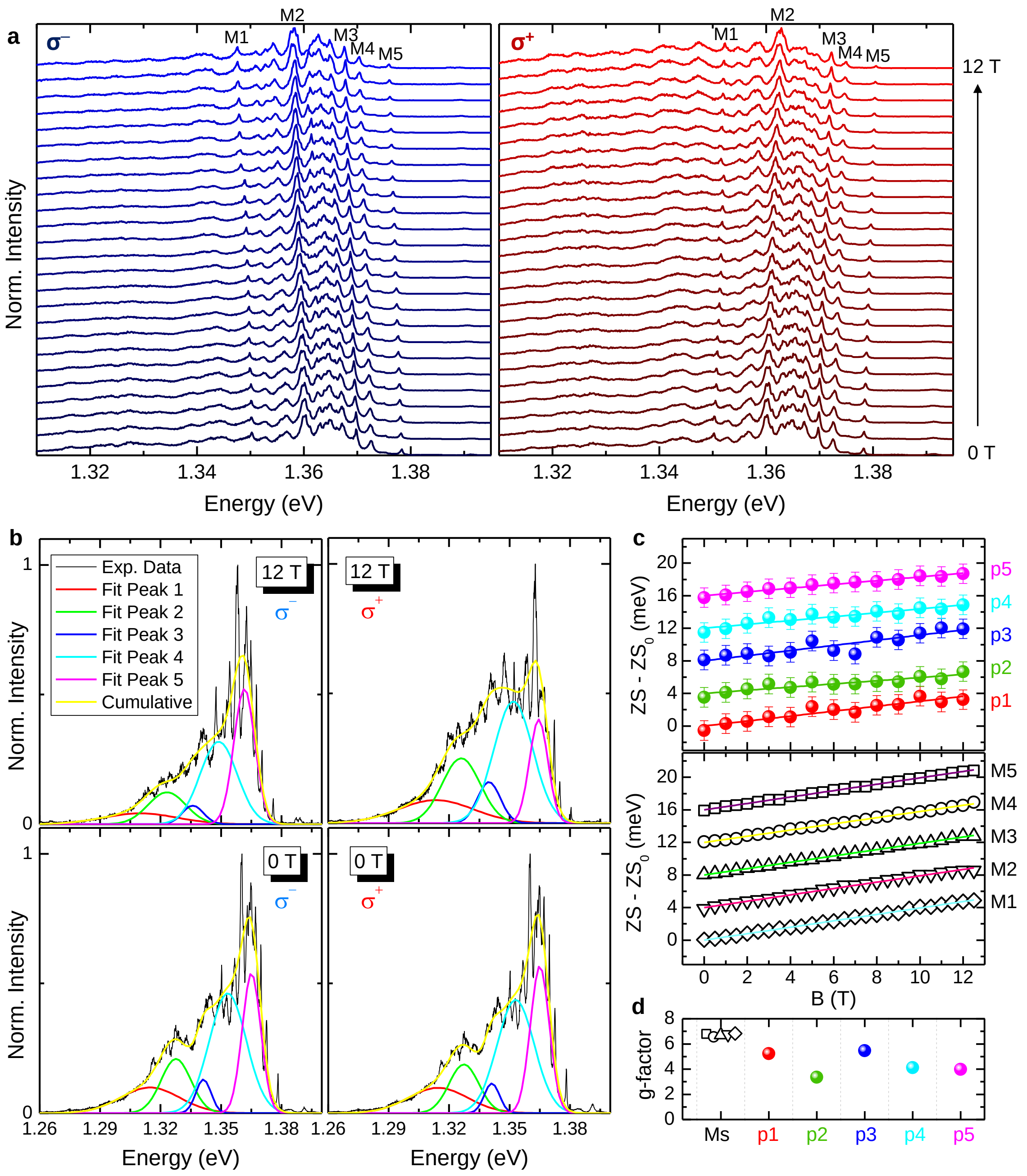

Figure 10.1: g-factor analysis of the moiré energy levels. **a** Polarisation-resolved magneto-μ-PL spectra acquired on HS1 with P_{exc} = 10 nW from 0 to 12 T in steps of 0.5 T. Five moiré narrow lines (Ms) are indicated. **b** Exemplifying spectra at 12 T and 0 T fitted by 5 gaussian peaks, for both the σ^- and σ^+ polarisations. **c** Top: ZS of the five gaussian peaks (ps) as a function of the magnetic field. The solid lines are linear fits to the data that provide the g-factors displayed in panel **d**. The intercept was subtracted from the data and they were stacked by 4 meV for ease of comparison. Bottom: Same for the 5 different moiré narrow lines. **d** Summary of the g-factors of both the narrow lines and the gaussian components. The error bars are smaller than (comparable to) the point size for the Ms (ps).

From this set of data, we first measured the g-factor of 5 different individual narrow lines (indicated as Ms), finding values between $+6.57$ and $+6.82$, see Fig. 10.1. We then fitted the whole set of data with multiple gaussians, as exemplified in panel **b** at 0 and 12 T.

Indeed, the 5 peaks feature a positive g-factor, similarly to the moiré single lines, but with smaller absolute value. This might possibly be attributed to the role played by exciton-exciton interactions.

These results are particularly significant since the similar values (sign and modulus) of the g-factor of the exciton manifold and of the single lines indicate a common electronic structure/origin of those recombination lines. In particular, it indicates that the narrow lines are indeed single moiré excitons giving rise to a gaussian distribution when a sufficiently high number of them is photogenerated. As a matter of fact, the exact nature of those narrow lines is a topic recently debated in the literature [1, 5].

Supplementary Note 11. Temperature-dependent g-factor of the MX/IX band

Figure 11.1 shows a series of magneto-μ-PL spectra recorded with an excitation power of 75 μW at different magnetic fields for T=210, 160 and 100 K, panels (a), (c) and (e), respectively. The spectra were recorded with opposite circular polarisation filtering (σ^+ and σ^-) and the ZS was then derived. The ZS dependence on B is shown in panels (b), (d) and (f) for T=210, 160 and 100 K, respectively. At the considered temperatures, g is negative and corresponds to the gyromagnetic factor of the free interlayer exciton $g_{\mathrm{exc,IX}}$. The values found for different Ts are shown in the corresponding panels and do not change appreciably with temperature. As discussed in the main text, the $g_{\mathrm{exc,IX}}$ sign and absolute value can be ascribed to the avoided action of the moiré potential determined by the T-induced MX de-trapping. Indeed, $g_{\mathrm{exc,IX}}$ can be estimated by considering the separate contribution of electrons and holes to the IX gyromagnetic factor (see Eq. (5) in the main text).

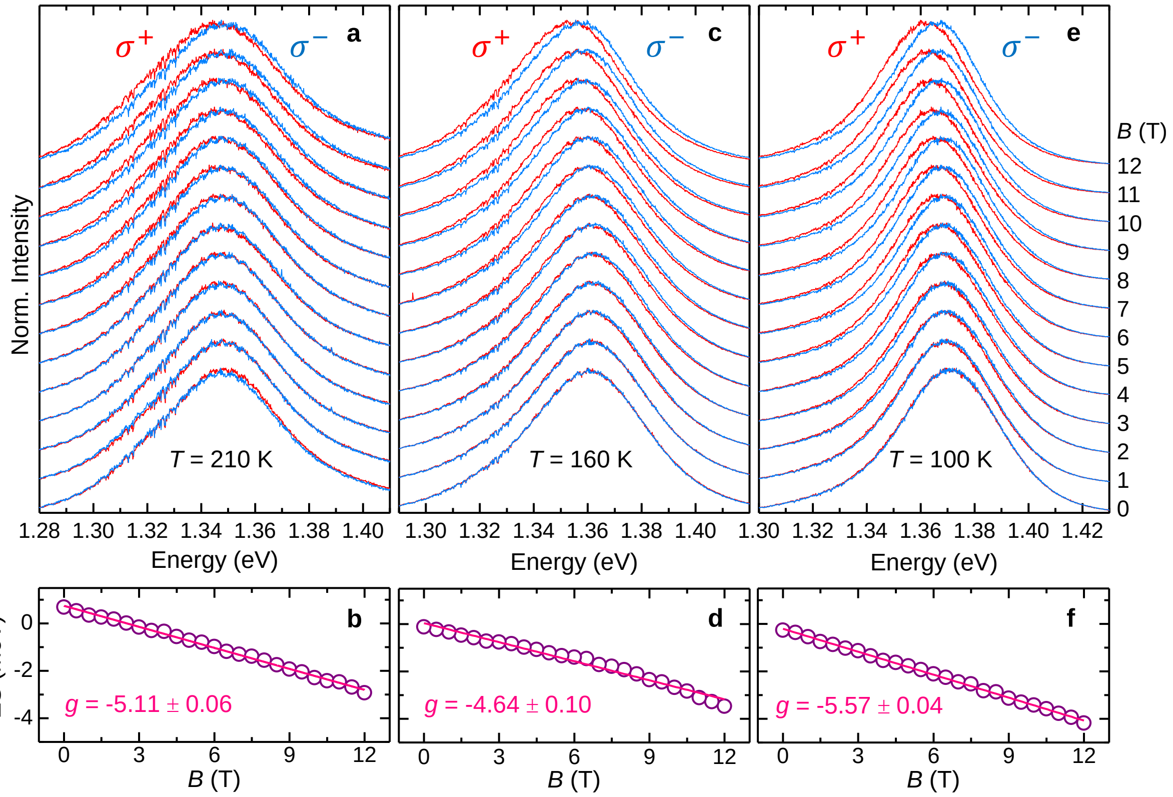

Figure 11.1: a Helicity-resolved normalised μ-PL spectra *vs* magnetic field at T = 210 K. **b** ZS of the band, from which the displayed g-factor was obtained through a linear fit. **c-d** Same as panels **a-b** for T = 160 K. **e-f** Same as panels **a-b** for T = 100 K. It should be noticed that the standard deviation associated to each fit clearly underestimates the real error on the g-factor. The latter is in fact a bit dependent on the exact sample position where the measurements are taken. By staying at the maximum field (12 T) and changing position, we observed ZS variations within 20-25 %. The results of panels **b**, **d** and **f** thus show that within the position-related uncertainty, a similar g-factor of $\approx +5$ is found at elevated temperatures. The same excitation power P_{exc} = 75 μW (focused via a 100× objective with NA = 0.8) was used for all the sets of data.

To further prove the localised-to-delocalised transition of interlayer excitons with temperature, we recorded magneto-μ-PL spectra at various intermediate temperatures, where the MX and IX coexist. However, disentangling the contribution of the overlapping MX and IX bands in the spectrum is not feasible, especially considering the necessity of using high laser powers to attain a sizable PL signal. This inevitably leads to a prevailing signal form the free IX band. We thus performed high-field (16 T) magneto-μ-PL measurements at a relatively high T of 80

K aimed at highlighting the MX component with respect to the free IX band. To do so, we employed very low laser powers of 10 nW. The results are shown in Fig. 11.2. We acquired the

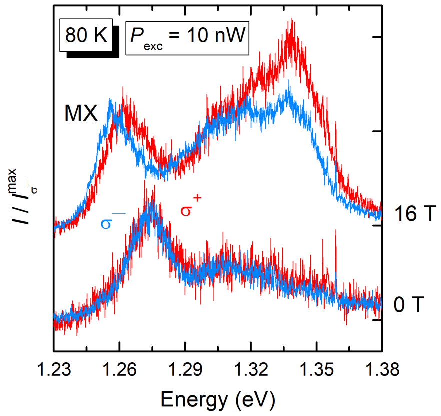

Figure 11.2: Polarisation-resolved magneto-μ-PL spectra acquired at 0 and 16 T on HS1 with a power as low as 10 nW, in order to highlight the MX contribution. The low energy peak, attributed to the MX, shows a clear positive Zeeman splitting. At each field, the MX peak of the σ^- component is normalised to 1.

spectra at 0 T and 16 T (a whole sweep was not feasible because of the very low PL signal due to the low power and high T employed) and observed a clear splitting of the MX band at 16 T, from which we derive an estimate of the g-factor of about +6. The data agree with previous assignments demonstrating that the positive gyromagnetic factor for MX is indeed found also at 80 K.

Supplementary Note 12. Power studies of the MX/IX band at 16 T and low T

Here we discuss additional magneto-μ-PL measurements on HS1 and HS2 employing a fixed magnetic field B of 16 T, with P_{exc} values ranging over five orders of magnitude. The results are shown in Fig. 12.1. With increasing P_{exc} (or equivalently $n_{\mathrm{e-h}}$), the positive Zeeman splitting (*i.e.*, $E^{\sigma^+} > E^{\sigma^-}$) of the narrow emission lines related to various moiré-confined excitons (see, *e.g.*, the narrow lines highlighted by the black circles at the lowest P_{exc}) becomes nearly zero as the PL band broadens and eventually turns negative on the high energy side of the μ-PL spectrum at the highest P_{exc} used. Thus, the high density of excitons achieved for large P_{exc} leads to an increased intercell exciton hopping [21], and to an ensuing sizable contribution from de-trapped moiré excitons to the emission spectrum that reflects in the change of lineshape (the appearance of a high energy contribution can be noticed) and especially in the change of sign of g_{exc}. This can be noticed especially for HS1. For HS2, the g-factor at high power is negative but small, presumably due to a coexistence of localised states and free excitons at the largest power we used.

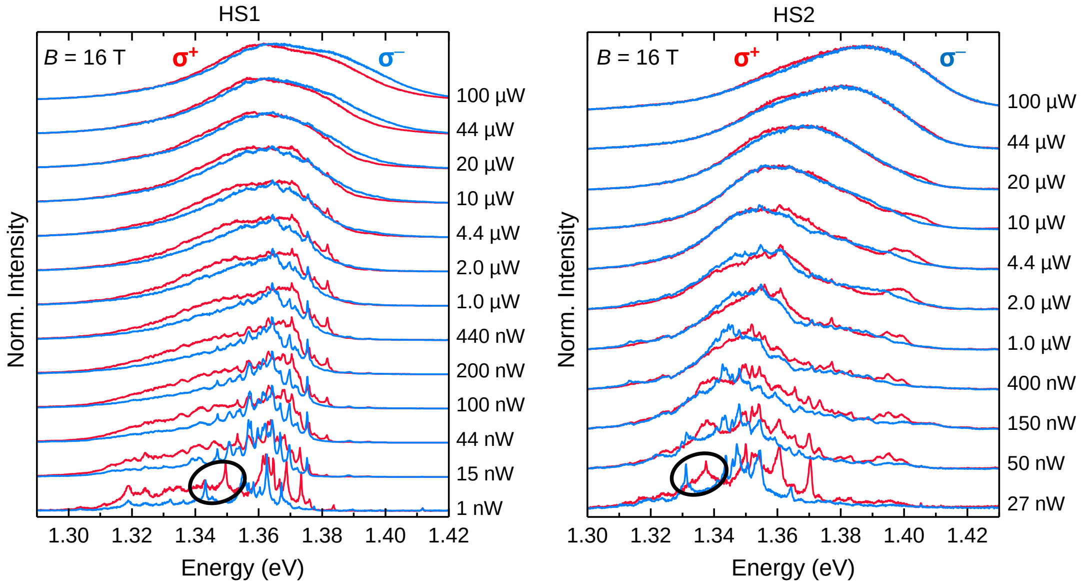

Figure 12.1: Polarisation-resolved μ-PL power studies performed at 10 K under a 16 T magnetic field for both HS1 and HS2. At low powers, several Zeeman-split narrow lines can be observed, such as those highlighted by the black circle. At high powers, a change in the sign of the Zeeman splitting can clearly be noticed.

References

[1] F. Mahdikhanysarvejahany, D. N. Shanks, M. Klein, Q. Wang, M. R. Koehler, D. G. Mandrus, T. Taniguchi, K. Watanabe, O. L. Monti, B. J. LeRoy, and J. R. Schaibley, *Localized Interlayer Excitons in $MoSe_2/WSe_2$ Heterostructures without a Moiré Potential*, ArXiv e-prints p. arXiv:2203.08052 (2022).

[2] E. Liu, E. Barré, J. van Baren, M. Wilson, T. Taniguchi, K. Watanabe, Y.-T. Cui, N. M. Gabor, T. F. Heinz, Y.-C. Chang, and C. H. Lui, *Signatures of moiré trions in $MoSe_2/WSe_2$ heterobilayers*, Nature **594**, 46 (2021).

[3] T. Wang, S. Miao, Z. Li, Y. Meng, Z. Lu, Z. Lian, M. Blei, T. Taniguchi, K. Watanabe, S. Tongay, D. Smirnov, and S.-F. Shi, *Giant Valley-Zeeman Splitting from Spin-Singlet and Spin-Triplet Interlayer Excitons in $WoSe_2/MoSe_2$ Heterostructure*, Nano Lett. **20**, 694 (2020).

[4] F. Mahdikhanysarvejahany, D. N. Shanks, C. Muccianti, B. H. Badada, I. Idi, A. Alfrey, S. Raglow, M. R. Koehler, D. G. Mandrus, T. Taniguchi, K. Watanabe, O. L. A. Monti, H. Yu, B. J. LeRoy, and J. R. Schaibley, *Temperature dependent moiré trapping of interlayer excitons in $MoSe_2$-WSe_2 heterostructures*, npj 2D Mater. Appl. **5**, 67 (2021).

[5] Z. Li, X. Lu, D. F. C. Leon, Z. Lyu, H. Xie, J. Hou, Y. Lu, X. Guo, A. Kaczmarek, T. Taniguchi, K. Watanabe, L. Zhao, L. Yang, and P. B. Deotare, *Interlayer Exciton Transport in $MoSe_2/WSe_2$ Heterostructures*, ACS Nano **15**, 1539 (2021).

[6] H. Kim, D. Dong, Y. Okamura, K. Shinokita, K. Watanabe, T. Taniguchi, and K. Matsuda, *Dynamics of Moiré Trion and Its Valley Polarization in a Microfabricated $WSe_2/MoSe_2$ Heterobilayer*, ACS Nano **17**, 13715 (2023).

[7] B. Miller, A. Steinhoff, B. Pano, J. Klein, F. Jahnke, A. Holleitner, and U. Wurstbauer, *Long-Lived Direct and Indirect Interlayer Excitons in van der Waals Heterostructures*, Nano Lett. **17**, 5229 (2017).

[8] M. Brotons-Gisbert, H. Baek, A. Campbell, K. Watanabe, T. Taniguchi, and B. D. Gerardot, *Moiré-Trapped Interlayer Trions in a Charge-Tunable $WSe_2/MoSe_2$ Heterobilayer*, Phys. Rev. X **11**, 031033 (2021).

[9] J. Wang, J. Ardelean, Y. Bai, A. Steinhoff, M. Florian, F. Jahnke, X. Xu, M. Kira, J. Hone, and X.-Y. Zhu, *Optical generation of high carrier densities in 2D semiconductor heterobilayers*, Sci. Adv. **5**, eaax0145 (2019).

[10] K. Tran, G. Moody, F. Wu, X. Lu, J. Choi, K. Kim, A. Rai, D. A. Sanchez, J. Quan, A. Singh, J. Embley, A. Zepeda, M. Campbell, T. Autry, T. Taniguchi, K. Watanabe, N. Lu, S. K. Banerjee, K. L. Silverman, S. Kim, E. Tutuc, L. Yang, A. H. MacDonald, and X. Li, *Evidence for moiré excitons in van der Waals heterostructures*, Nature **567**, 7746, 71–75 (2019).

[11] W. Li, X. Lu, J. Wu, and A. Srivastava, *Optical control of the valley Zeeman effect through many-exciton interactions*, Nat. Nanotechnol. **16**, 148 (2021).

[12] K. L. Seyler, P. Rivera, H. Yu, N. P. Wilson, E. L. Ray, D. G. Mandrus, J. Yan, W. Yao, and X. Xu, *Signatures of moiré-trapped valley excitons in $MoSe_2/WSe_2$ heterobilayers*, Nature **567**, 66 (2019).

[13] W. Li, X. Lu, S. Dubey, L. Devenica, and A. Srivastava, *Dipolar interactions between localized interlayer excitons in van der Waals heterostructures*, Nat. Mater. **19**, 624 (2020).

[14] J. Choi, M. Florian, A. Steinhoff, D. Erben, K. Tran, D. S. Kim, L. Sun, J. Quan, R. Claassen, S. Majumder, J. A. Hollingsworth, T. Taniguchi, K. Watanabe, K. Ueno, A. Singh, G. Moody, F. Jahnke, and X. Li, *Twist Angle-Dependent Interlayer Exciton Lifetimes in van der Waals Heterostructures*, Phys. Rev. Lett. **126**, 047401 (2021).

[15] H. Baek, M. Brotons-Gisbert, Z. X. Koong, A. Campbell, M. Rambach, K. Watanabe, T. Taniguchi, and B. D. Gerardot, *Highly energy-tunable quantum light from moiré-trapped excitons*, Sci. Adv. **6**, 37, eaba8526 (2020).

[16] M. Troue, J. Figueiredo, L. Sigl, C. Paspalides, M. Katzer, T. Taniguchi, K. Watanabe, M. Selig, A. Knorr, U. Wurstbauer, and A. W. Holleitner, *Extended Spatial Coherence of Interlayer Excitons in $MoSe_2/WSe_2$ Heterobilayers*, Phys. Rev. Lett. **131**, 036902 (2023).

[17] P. Parzefall, J. Holler, M. Scheuck, A. Beer, K.-Q. Lin, B. Peng, B. Monserrat, P. Nagler, M. Kempf, and T. Korn, *Moiré phonons in twisted $MoSe_2$-WSe_2 heterobilayers and their correlation with interlayer excitons*, 2D Materials **8**, 035030 (2021).

[18] H. Kim, K. Aino, K. Shinokita, W. Zhang, K. Watanabe, T. Taniguchi, and K. Matsuda, *Dynamics of Moiré Exciton in a Twisted $MoSe_2/WSe_2$ Heterobilayer*, Adv. Optical Mater. **11**, 2300146 (2023).

[19] M. R. Rosenberger, H.-J. Chuang, M. Phillips, V. P. Oleshko, K. M. McCreary, S. V. Sivaram, C. S. Hellberg, and B. T. Jonker, *Twist Angle-Dependent Atomic Reconstruction and Moiré' Patterns in Transition Metal Dichalcogenide Heterostructures*, ACS Nano **14**, 4550 (2020).

[20] F. Wu, T. Lovorn, and A. H. MacDonald, *Theory of optical absorption by interlayer excitons in transition metal dichalcogenide heterobilayers*, Phys. Rev. B **97**, 035306 (2018).

[21] S. Brem and E. Malic, *Bosonic Delocalization of Dipolar Moiré Excitons*, Nano Lett. **23**, 4627 (2023).
